# Acute Neurological Aggravation Caused by Intratumoral Hemorrhage of a Cervical Dumbbell Schwannoma: Report of a Rare Case and Literature Review

**DOI:** 10.7759/cureus.34682

**Published:** 2023-02-06

**Authors:** Kotaro Sakashita, Masao Koda, Hiroshi Takahashi, Toru Funayama, Masashi Yamazaki

**Affiliations:** 1 Department of Orthopaedic Surgery, Faculty of Medicine, University of Tsukuba, Tsukuba, JPN

**Keywords:** dumbbell tumor, cervical tumor, intratumoral hemorrhage, neurilemmoma, schwannoma

## Abstract

Schwannomas are one of the most common types of primary intraspinal tumors. We report a rare case of neurological aggravation due to the intratumoral hemorrhage of a cervical schwannoma. A 65-year-old man presented with lower extremity weakness developing gradually. Tumor resection was performed one week after neurological aggravation occurred. After surgery, he recovered dramatically. There are vascular and mechanical hypotheses for the etiology of intratumoral hemorrhage of schwannoma. In the present case, falling and antiplatelet drugs may have caused the intratumoral hemorrhage. Optimal surgical timing remains controversial. Some reports reveal patients recovered well after urgent surgery. However, even if urgent surgery is performed, some have neurological sequelae. Others reveal patients recovered well after elective surgery without any sequelae. Because previous reports reveal the surgical procedure may damage the spinal cord, urgent surgery may not be compulsory and elective surgery may be a better treatment option. Further investigation is needed to clarify the etiology and optimal timing for surgical treatment of intratumoral hemorrhage.

## Introduction

Schwannomas are one of the most common types of primary intraspinal tumors, accounting for 30% of primary spinal tumors. They are usually asymptomatic or sometimes can cause radicular pain and slowly progressive neurological deficits [1,2]. Due to rare symptoms, delayed diagnosis is common and sometimes tumors are diagnosed incidentally on imaging.

In cases of spinal tumors, acute onset paralysis is rarely caused by an intratumoral hemorrhage; common types of spinal tumors are ependymoma, cavernous, and hemangioblastoma. Intratumoral hemorrhage of a schwannoma is rare [1]. Even when urgent surgery is performed, some patients have neurological sequelae. Therefore, the optimal timing of surgery remains unclear. Here, we present a rare case of acute neurological aggravation caused by intratumoral hemorrhage of a cervical schwannoma and discuss the etiology and optimal timing for its surgical management.

## Case presentation

**Figure 1:**
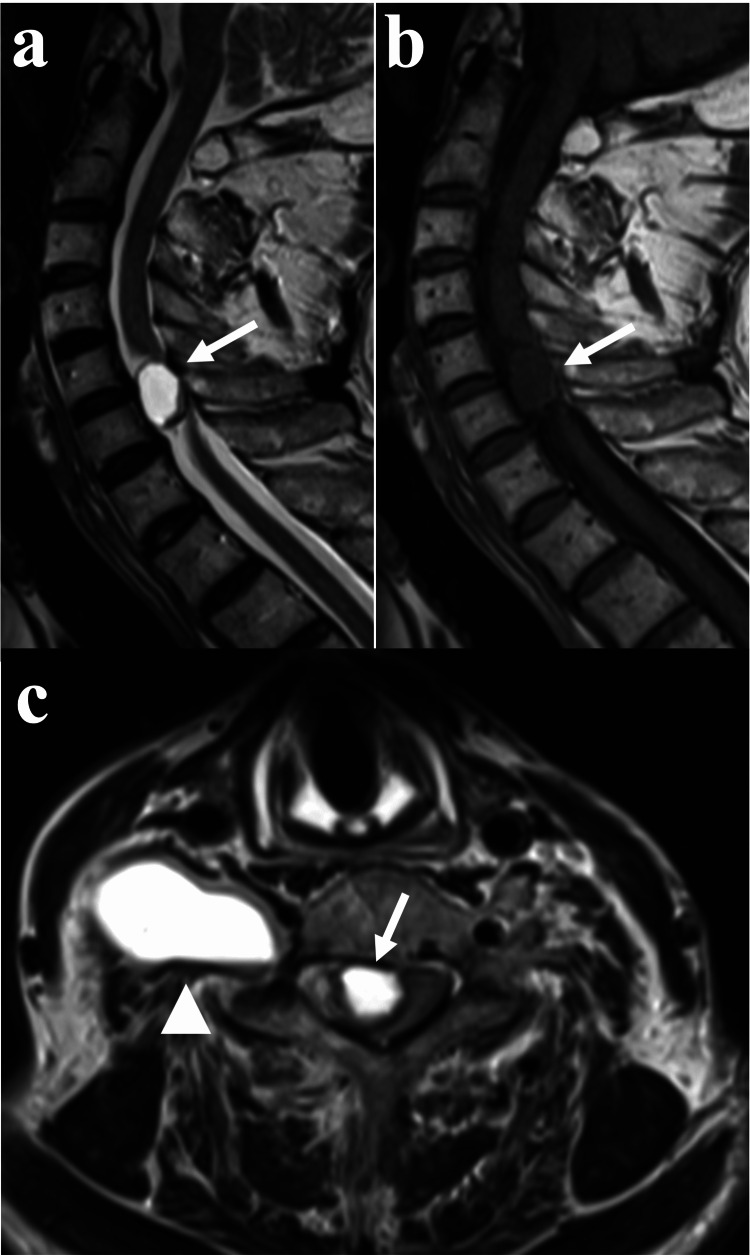
MRI of the cervical spine (a) Sagittal T2-weighted image showing a hyperintense area at the C5-6 level. The arrow indicates the hypointense lesion attached to the tumor. (b) Sagittal T1-weighted image showing an isointense area. The arrow indicates the isointense lesion attached to the tumor, indicating a hematoma. (c) Axial T2-weighted image showing a hyperintense lesion severely compressing the spinal cord and a hypointense to isointense lesion (arrow). The hyperintense lesion is spread anteriorly through the foramen and anterior part including fluid-fluid level (arrow head), indicating hemorrhage.

A 65-year-old Japanese man presented with difficulty grasping, and numbness in his right upper limb for the previous two months. He had been referred to a local hospital and was diagnosed with an old cerebral infarction and prescribed antiplatelet drugs. However, he was finally referred to the neurology department of another hospital due to acute progression in difficulty in standing after suffering a fall. Magnetic resonance imaging (MRI) revealed a cervical spinal dumbbell tumor, and the patient was referred to our hospital for surgery (Figure 1). An Eden type 2 dumbbell tumor had invaded the spinal canal at the C5-6 level, severely compressing the spinal cord. We suspected acute deterioration of myelopathy caused by intratumoral hemorrhage, which might have enlarged the tumor volume and compressed the spinal cord, because the tumor had some fluid-fluid level on MRI (Figure 1). The patient exhibited paralysis of his lower extremities classified on the modified Frankel scale as C2 and bladder and bowel dysfunction (BBD). Even though the patient was advised strict bed rest, the weakness in his lower extremities progressed to modified Frankel scale classification C1 four days after admission. Therefore, partial tumor resection was performed.

**Figure 2:**
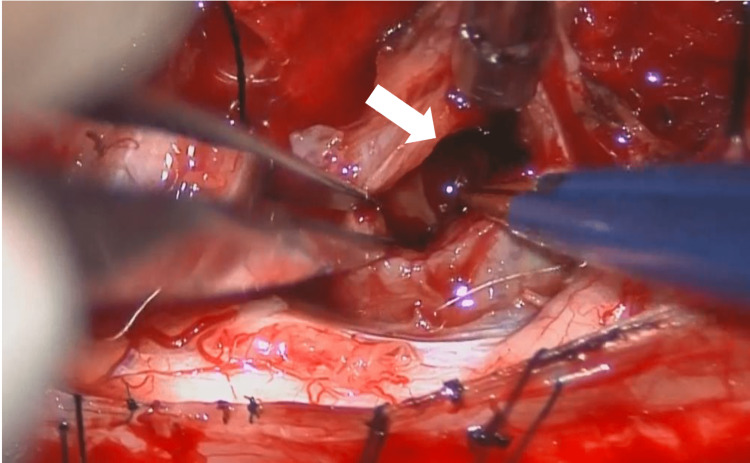
Intraoperative image, with hematoma (arrow) visible after the incision of the capsule

After the incision of the dura, we found that the tumor was derived from the C6 ventral root, which was sacrificed to extirpate the tumor invading the spinal canal, and included a hematoma indicating that intratumoral hemorrhage had occurred (Figure 2). As much of the tumor as we could see from the posterior approach was excised.

**Figure 3:**
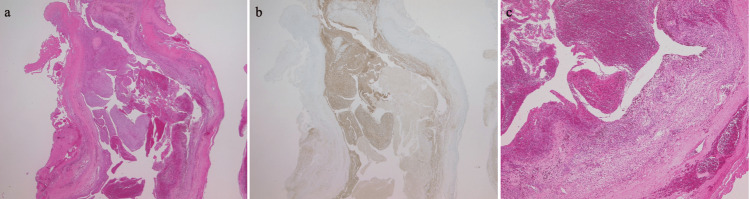
Photomicrographs (a) Hematoxylin and eosin staining (×12.5) showing both hypercellular (Antoni A) and hypocellular (Antoni B) regions composed of spindle cells with hemorrhage. (b) Immunohistochemistry for S100 (×12.5) is positive for tumor cells and negative for hemorrhage and capsule. (c) High-power magnification of hematoxylin and eosin staining (×40) shows hemosiderin deposition and hemorrhage within a sparse (Antoni B) area of the tumor.

The histopathology of the tumor was consistent with a benign schwannoma including the area of hemorrhage and hemosiderin deposition (Figure 3). After the surgery, the patient fortunately had no motor deficit. He had clearly recovered within four months and could ambulate without aid. Two years after the surgery, he had been progressing satisfactorily and returned to work.

## Discussion

This case of acute neurological aggravation was caused by a compressive tumor enlarged by an intratumoral hemorrhage. Hemorrhage of benign neural tumors is rare, although there are some reports of spinal cord tumors presenting with subarachnoid or intratumoral hemorrhage. Because tumor-associated arachnoid adhesions prevent the subarachnoid spread of the blood, the bleeding in the present case was localized, leading to tumor mass expansion [1].

To our knowledge, 13 reports describing intratumoral hemorrhage in schwannomas have been published to date (11 reports in English and 2 in Japanese). As such, this is the first case of a dumbbell tumor presenting with intratumoral hemorrhage. There are vascular and mechanical hypotheses about the etiology of intratumoral hemorrhage of a schwannoma. The vascular hypothesis is that hyalinized ecstatic vessels inside the schwannoma may undergo spontaneous thrombosis leading to distal necrosis and hemorrhage. The mechanical hypothesis is that traction on the tumor vasculature during movement results in the disruption of blood vessels and hemorrhage [3]. Additionally, another etiology has been postulated and is associated with anticoagulant/platelet drugs, normal vaginal delivery, intense sneezing, and nerve root torsion [4-8].

In our case, it was found, histopathologically, that there were hypocellular areas composed of proliferated spindle cells with a hemorrhagic lesion and hemosiderin deposition within the mass. This suggests that some parts of the tumor tissues were so fragile that minor hemorrhage might have occurred as a result of falling, and antiplatelet drugs might have exacerbated the hemorrhage leading to massive intratumoral hemorrhage. When the patient was admitted to our hospital, he had paralysis of the lower extremities, classified as C2 on the modified Frankel scale, and seven days had passed since the onset of the paralysis without any rest restriction. Because we have seen patients with neurological deterioration who recovered from their paralysis with strict bed rest, we closely monitored the patient [9]. At that time, we suspected that if instability was related to neurological aggravation, bed rest would be effective, and we could eventually avoid complications of surgery due to our surgical manipulation. For these reasons, we first monitored the patient, but since acute spinal cord compression caused by intrautumoral hemorrhage was the main cause of acute neurological aggravation, strict bed rest was not effective and we decided to perform surgery.

In our literature review, urgent tumor resection was performed as observed in nine reports [1,2,7,10-15], elective surgery reported by two [4,16], with no record in another two [8,17] (Table 1). Complete recovery was achieved in five of nine patients who underwent urgent surgery, whereas four had neurological sequelae, such as ambulation with aid, no improvement in BBD and quadriplegia with respiratory paralysis [1,4,10]. After elective surgery, a patient recovered completely and another needed some aid during ambulation [4,13]. There are some other reports of elective surgery for the treatment of intratumoral hemorrhage in spinal tumors. A previous case report revealed natural regression of an intratumoral hemorrhage of the spinal schwannoma [13]. The other report revealed that tumor removal surgery presents a risk of additional damage to the spinal cord after the acute onset of sensorimotor deficits [18].

**Table 1:** Reported cases of intratumoral hemorrhage of schwannoma BBD: bladder and bowel dysfunction, NR: not recorded

First author/year	Age/sex	Location	Predisposing factor	Symptoms	Onset	Timing of surgery	Results
Cohen/2000 [1]	52/Male	T11-12	Minor trauma	Paraplesia, urinary incontinence, back pain	Acute	Emergent	Ambulant with aid, self-catheterization
Jung/2019 [2]	37/Male	C2-3	Spontaneous	Quadreparesis, neck pain	Acute	Emergent	Fully recovered
Ichinose/2009 [4]	64/Male	T12-L1	Spontaneous (anticoagulant therapy)	Paraparesis, dysesthesia, BBD	Acute	Elective	Fully recovered
Liang/2000 [7]	60/Male	C2-3	intense sneezing	Hemiplegia, sensory disturbance, neck pain	Acute	Emergent	Fully recovered
Jenkins III/2015 [8]	62/Male	L2-3	Spontaneous (torsion)	Muscle weakness, sensory disturbance	Acute	NR	Fully recovered
Rahyssalim/2019 [10]	38/Female	T10-12	Spinal manipulation	Paraplesia, numbness, BBD	Subacute	Emergent	Ambulant with aid, no improvement in BBD
Kato/2010 [11]	27/Male	C2-4	Spontaneous	Quadriplegia, respiratory paralysis	Acute	Emergent	No improvement
Gandhoke/2018 [12]	38/Male	C2-4	Spontaneous	Spastic quadreparesis, neck pain	Acute	Emergent	Fully recovered
Ciapetta/2008 [13]	44/Female	C2	Spontaneous	NR	Acute	Emergent	Fully recovered
Sahoo/2015 [14]	44/Male	C3-5	Spontaneous	Quadriparesis, neck pain	Acute	Emergent	Fully recovered
Uemura/1998 [15]	58/Female	T12	Spontaneous	Paraparesis, hypalgesia	Acute	emergent	NR
Ito/2019 [16]	58/Male	L4-5	Spontaneous	Muscle weakness, sensory disturbance, back pain, leg pain	Acute	Elective	Fully recovered
Kukreja/2014 [17]	47/Male	T12-L1	Spontaneous	Seizure, leg pain	Subacute	NR	Fully recovered

We cannot draw a clear conclusion for the optimal timing of the surgical intervention for intratumoral hemorrhage of spinal schwannoma from our literature review. We cannot ignore the fact that there were patients who had neurological sequelae caused by intratumoral hemorrhage even when urgent tumor resection was performed. In our case, we observed the patient closely and decided to perform surgery a week after the acute neurological deterioration occurred, resulting in dramatic recovery. We believed that paralysis grade worse than the modified Frankel classification of C1 required emergency surgery. According to the American Spinal Injury Association (ASIA) Impairment Scale (AIS), the modified Frankel classification of C1 is equivalent to AIS C. Patients with AIS C or worse have been included in many of the studies of surgery for spinal cord injury [19,20]. For this reason, we believed that the modified Frankel classification of C1 is important. Patients should be observed carefully, and elective surgery to avoid complications should be planned. However, whether urgent tumor resection or elective surgery for acute neurological deterioration caused by intratumoral hemorrhage results in better outcomes remains controversial. Further investigation is needed to clarify the etiology and determine the optimal timing for surgical treatment of intratumoral hemorrhage.

## Conclusions

Here, we have reported a rare case of neurological aggravation due to the intratumoral hemorrhage of a cervical schwannoma. There are vascular and mechanical hypotheses for the etiology of intratumoral hemorrhage of the schwannoma; in addition, the fall and antiplatelet drugs may have caused intratumoral hemorrhage. Tumor resection was performed one week after neurological aggravation occurred, resulting in dramatic recovery. Although we cannot draw a clear conclusion for the optimal timing of surgical intervention for intratumoral hemorrhage of the spinal schwannoma, the present report revealed that patients should be closely monitored, and surgery may be an option to avoid complications.
